# Pertussis epidemiological pattern and disease burden in Brazil: an analysis of national public health surveillance data

**DOI:** 10.1080/21645515.2019.1634991

**Published:** 2019-07-24

**Authors:** Eliana Nogueira Castro De Barros, Altacilio Aparecido Nunes, Ariane De Jesus Lopes De Abreu, Bárbara Emoingt Furtado, Otavio Cintra, Monica Act Cintra, Eduardo Barbosa Coelho

**Affiliations:** a GSK, Rio de Janeiro, RJ, Brazil; b Ribeirão Preto Medical School, University of São Paulo, Brazil; c Shift Gestão de Serviços, Rio de Janeiro, RJ, Brazil; d GSK, Wavre, Belgium

**Keywords:** Brazil, epidemiology, vaccination, Bordetella pertussis, Tdap

## Abstract

**Objective:** We described pertussis epidemiological trends in Brazil between 2010 and 2015. We also assessed tetanus, diphtheria and acellular pertussis (Tdap) vaccine coverage among pregnant women from 2014, the year of the introduction of Tdap maternal immunization recommendation in Brazil, to 2016.

**Methods:** Epidemiological data for incidence, prevalence, hospitalization, mortality, and maternal vaccination coverage were calculated based on the Brazilian public surveillance databases.

**Results:** The epidemiological data analysis results showed that the pertussis average incidence rate (IR) was 2.19/100,000 inhabitants for all ages, with a peak in 2014 (4.03/100,000 inhabitants) and highest incidence in <1-year-old children (IR = 175.20/100,000). 97.6% of pertussis deaths (405/415) were in <1-year-old children. Maternal immunization coverage was 9.2% in 2014, 40.4% in 2015, and 33.8% in 2016.

**Conclusions:** Pertussis incidence and pertussis-related deaths increased in Brazil from 2010 to 2014 and decreased in 2015. In the two years, 2015 and 2016 that followed the NIP recommendation, Tdap vaccination coverage of pregnant women was low and varying from region to region. More efforts and national plans would help increase awareness and maternal immunization coverage.

## Introduction

Pertussis (whooping cough) is a highly contagious respiratory disease^^1^^^,^^2^ caused by the human-restricted pathogen *Bordetella pertussis* (*B. pertussis*).^3^ The infection is transmitted with droplets spread by coughing, sneezing, or speaking.^1^^,^^4^ The incubation period lasts 7 to 10 days and then the illness develops for 6 to 12 weeks through three phases: catarrhal, paroxysmal, and convalescent. Its typical clinical course in unimmunized children begins with respiratory symptoms and a gradual worsening of the cough, progressing to the characteristic paroxysms followed by whooping and post-tussive vomiting.^1^ Apnea and cyanosis are among the initial symptoms in children <1 years of age^5^ that may lead to seizures, and also severe pulmonary hypertension, resulting in death.^1^ If untreated, the infected person is highly contagious for up to three weeks.^6^^,^^7^ The disease is transmitted to children <1 year of age especially by their mothers, and to a lesser extent by their fathers and grandparents.^8^

Immunity to pertussis can be acquired after the disease or after vaccination, however the protection wanes over time.^9^^,^^10^ The Brazilian National Immunization Program (NIP) recommends immunization with diphtheria, tetanus and whole cell pertussis vaccine (DTP) at two, four, six and 15 months of age, with a booster between four and six years of age.^11^ Furthermore, because maternal immunization has been shown to be effective in protecting babies from pertussis until the completion of their primary vaccination,^5^^,^^12^ the Brazilian NIP has been recommending, since 2014, booster with reduced antigen content of tetanus, diphtheria, and acellular pertussis vaccine (Tdap) for pregnant women starting from the 27th week of gestation. In Brazil, all population has free of charge vaccination offered by the NIP. Updated recommendations issued in 2017 state that vaccination should start from the 20th week of each pregnancy. Women who missed the opportunity to be vaccinated during pregnancy should receive a dose of Tdap in the post-partum as early as possible (within less than 45 days post-partum).^11^

Despite achieving approximately 85% global coverage since 2010 with three DTP doses,^13^ a resurgence of pertussis in recent years was observed.^14^^,^^15^ Pertussis epidemiological pattern usually presents in cycles with incidence peaking every 3–5 years;^15^ however, in some countries, the pertussis incidence increases represented a true resurgence of the disease.^16^^,^^17^^,^^18^^,^^19^ In Brazil,^20^ analyzed surveillance data 2007–2014 showed that pertussis had re-emerged in Brazil. Of the confirmed cases, 34.5% were in patients aged ≤2 months of age, 22.4% were in patients aged from 3 to 6 months of age, and 21% in patients aged 7 months to 4 years old. The overall case fatality rate was 2.1%, and was higher in children <3 months of age (4.7%). The expected vaccination coverage of 95% with three DTP doses was reached only in nine of the 27 Brazilian states (26 states and the Federal district) in 2012, but this number was increased to 17 in 2013 and 21 in 2014.^20^

**Figure 1.**
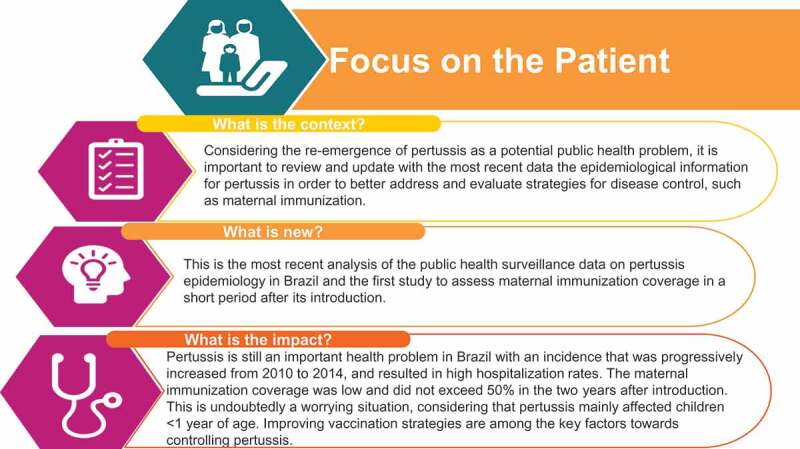
Relevance, aims and main findings of the study.

In this context, based on the fact that the surveillance of the evolution of pertussis epidemiology needs to be reviewed regularly and considering the re-emergence of pertussis as a potential public health problem for Brazil, it is important to examine and update with the most recent data the epidemiological information for pertussis in Brazil in order to better address and evaluate strategies for disease control, such as maternal immunization. The aim of this study was: to describe pertussis epidemiological trends in Brazil between 2010 and 2015 based on the pertussis surveillance data of the national public health databases (see Figure 1). The current analysis included periods before and one year after Tdap maternal immunization recommendation by NIP in 2014.

## Results

Overall, 26,086 pertussis cases were reported in the period 2010–2015, 15,703 hospitalizations, and 415 deaths due to pertussis (Table 1). Children <1 year of age accounted for 61.9% (16,150/26,086) of all cases; 15.6% (4,060/26,086) were from 1 year to 4 years of age and 22.5% (5,876/26,086) were >4 years of age.

**Table 1.** Number of pertussis cases, hospitalizations and deaths, mean incidence rate per 100,000 inhabitants, prevalence rate per 100,000 inhabitants, and median case fatality rate, in Brazil and by region from 2010 to 2015.

		Hospitalizations due to pertussis	Deaths due to pertussis			Case fatality rate^b^	
	Pertussis cases N (%)	N (%)	All ages, N (% in all deaths)	Mean incidence rate^a^	Prevalence rate	All ages, median (%)	Children <1 year of age, mean (%)
**Brazil**	**26,086 (100)**	**15,703 (100)**	**415 (100)**	**2.19**	**13.01**	**1.90**^‡^	**3.2**
Southeast	11,506 (45)	6,111 (39)	175 (42)	2.28^†^	13.88^†^	1.60^‡^	3.3
Northeast	6,081 (24)*	4,142 (26)*	99 (24)*	1.81^†^	10.93^†^	1.75^‡^	2.6
South	5,254 (21)*	2,863 (18)*	49 (12)*	2.75	18.55	0.93^‡^	1.9
Midwest	1,930 (8)*	1,351 (9)*	52 (12)*	2.23^†^	13.58^†^	2.37^‡^	2.8
North	1,315 (2)*	1,236 (8)*	40 (10)*	1.67^†^	10.31^†^	2.30^‡^	5.7

Source data: SINAN,^21^ SIH, ^22^ SIM^23^

Footnote: ^a^average incidence per 100,000 inhabitants; ^b^calculated as follows: number of deaths/number of pertussis cases in each region; *p < 0.05 (each of the Brazilian regions compared to the Southeast Region; X^2^ test); ^†^p < 0.05 (each of the Brazilian regions compared to the South Region; Student t-test for independent samples); ^‡^p < 0.05 (All ages compared to the children <1 year of age; Brazil and between each of the Brazilian regions; Student t-test for independent samples)

### Pertussis cases and incidence rates

The highest mean incidence rate, in the overall population (all ages) from 2010 to 2015, was reported in the South region (Table 1). The highest number of pertussis cases, pertussis-related hospitalizations, and deaths was reported in the Southeast (Table 1). The differences between proportions of pertussis cases, pertussis-related hospitalizations, and deaths were statistically significant lower in all comparisons between each region and the Southeast region that had the highest respective proportions. Five states had the highest absolute number of pertussis cases during the studied period: São Paulo (6,430), Espírito Santo (2,771), Paraná (2,389), Pernambuco (2,145) and Rio Grande do Sul (1,916).

**Figure 2.**
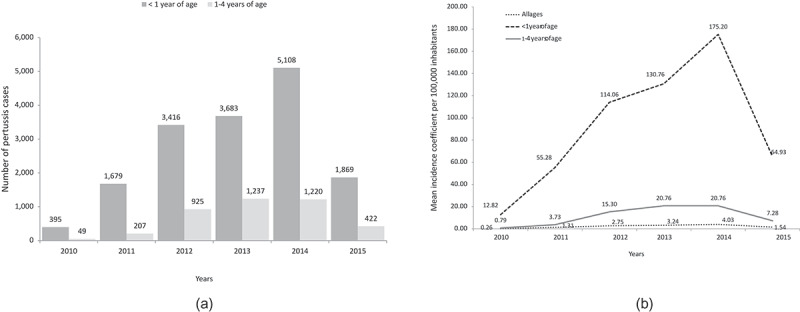
(a) Yearly pertussis cases by age group and (b) yearly mean incidence rate per 100, 000 inhabitants by age group and all ages. Source data: SINAN^24^

The number of pertussis cases in young children during the study period is shown in Figure 2(a). The mean incidence rate for the period 2010–2015 in the total population was 2.19 per 100,000 inhabitants (Table 1). From 2010 to 2014, there was a marked increase in pertussis incidence in children <1 year of age (from 12.82 per 100,000 inhabitants in 2010 to 175.20 in 2014) followed by a reduction in 2015 (Figure 2(b)).

**Figure 3.**
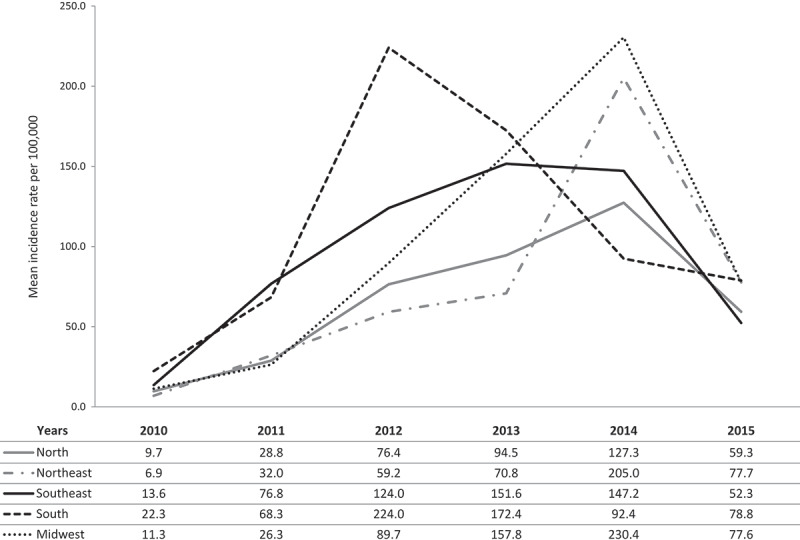
Pertussis mean incidence rate per 100, 000 children <1 year of age by region and year.

**Figure 4.**
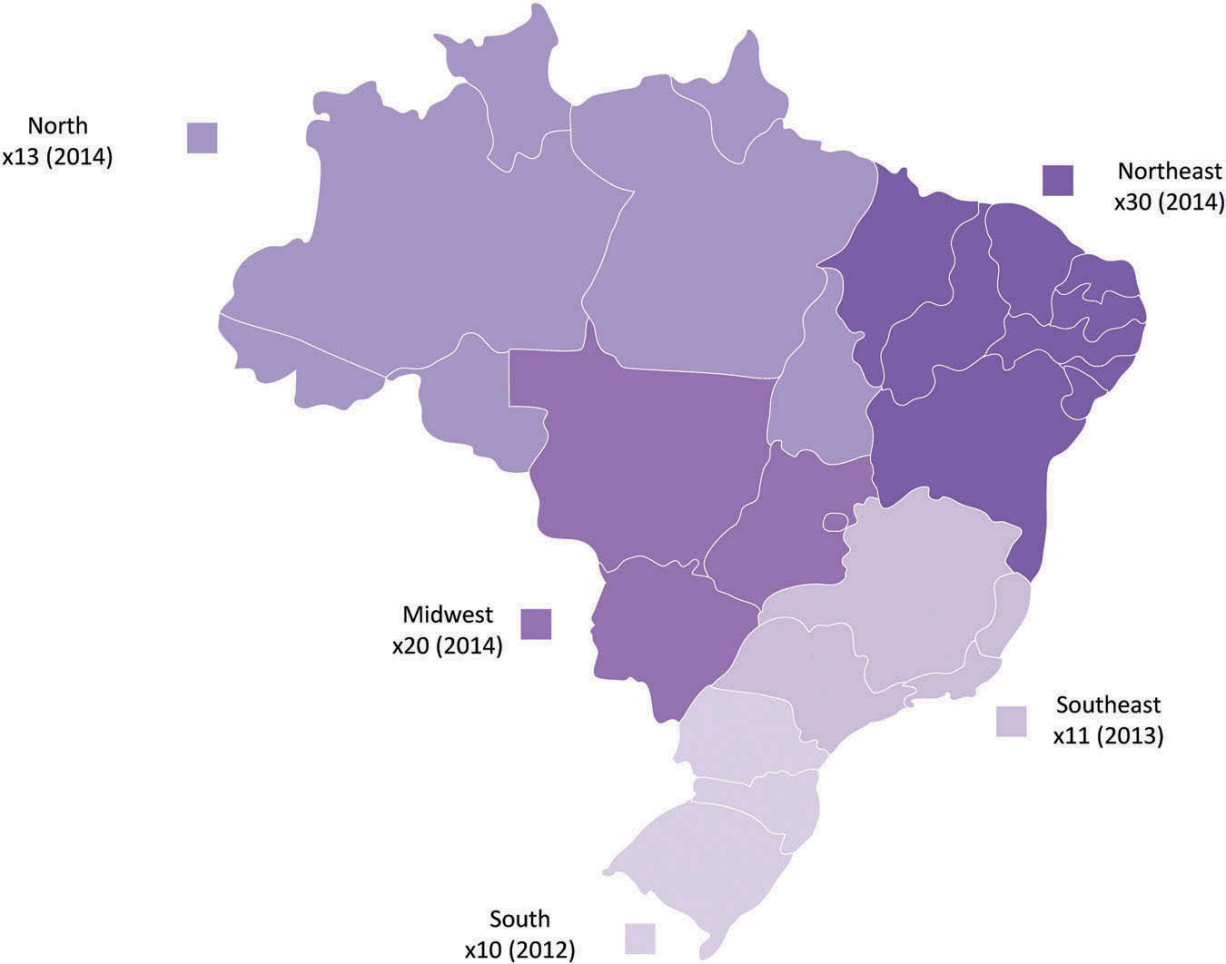
Map of Brazil, displaying, by region, the maximum increases in the incidence of pertussis infection in children <1 year of age to peak year, times increased from 2010 (peak year).

In all regions, the incidence of the disease in children <1 year of age was very high from 2011 to 2014 (Figure 3). The South region was the first to reach its highest incidence rate per 100,000 children <1 year of age, in 2012, followed by the Southeast region in 2013 – all other regions reached their highest incidence level in 2014 (Figures 3 & 4). Figure 4 graphically displays the maximum increases from 2010 in the incidence of pertussis infection in children <1 year age, by geographical region: the number in the parenthesis represents the year with the highest incidence (peak year) and the number before the parenthesis corresponds to the number of times that the incidence was increased from 2010 to the peak year.

### Hospitalizations and deaths

Overall 15,703 hospitalizations due to pertussis were reported (Table 1). Considering that each case of pertussis generated only one hospitalization episode, the percentages of hospitalizations for the disease in Brazil were 60.2% (15,703/26,086); North 94.0% (1,236/1,315), Midwest 70.0% (1,351/1,930), Northeast 68.1% (4,142/6,081), South 54.5% (2,863/5,254), and Southeast 53.1% (6,111/11,506).

**Figure 5.**
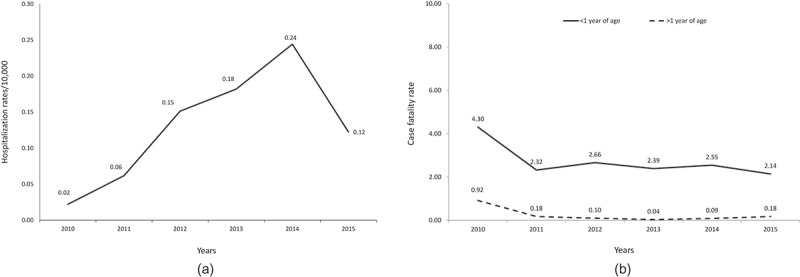
Number of (a) hospitalization rates and (b) case fatality rates due to pertussis in Brazil over years. Source data: (A) SIHSUS^25^ (B) SIM^26^

Hospitalizations due to pertussis gradually increased until 2014 and decreased in 2015 (Figure 5(a)). Deaths due to pertussis (Figure 5(b)), involved mainly children <1 year of age (97.6%, 405/415). Among the five geographical regions of Brazil, Midwest and North regions had the highest case fatality rate in the overall population (all ages) (*p* < .05); North region had the highest case fatality rate in children <1 year of age (Table 1).

### Maternal immunization: pertussis vaccine coverage

In the last quarter of 2014, Tdap vaccination was implemented nationally, yet the vaccination coverage was low. A gradual increase in vaccination coverage was observed in 2015 and 2016 in the South, Midwest, and North region. However, in the other two regions, Southeast and Northeast, although Tdap vaccination coverage was increased in 2015, it was decreased in 2016 (Table 2).

**Table 2.** Maternal vaccination coverage with Tdap in Brazil and by region from 2014 to 2016.

	Annual Tdap vaccination coverage (%) in pregnant women^a^		
	2014	2015	2016
**Brazil**	**9.2**	**40.4**	**33.8**
Southeast	10.3	50.7	32.4^‡^
Northeast	10.3	41.4^†^	36.3^‡^
South	6.3*	26.0^†^	30.0 ^‡^
Midwest	10.2	31.7^†^	45.7
North	5.1*	23.9^†^	28.3^‡^

Source data: SI-PNI^27^

Footnote: ^a^calculated by DATASUS based on their records as follows: number of women vaccinated/number of women in the target population X 100;^27^ *p < 0.05 (South and North Regions compared to the Southeast, Northeast and Midwest Regions; X^2^ test); ^†^p < 0.05 (each of the Brazilian regions compared to the Southeast Region; X^2^ test); ^‡^p < 0.05 (each of the Brazilian regions compared to the Midwest region; X^2^ test)

DISCUSSION

The present analysis of pertussis surveillance data from the Brazilian Ministry of Health (MoH) showed that from 2010 to 2015, the incidence of pertussis progressively increased in all Brazilian regions. The increase was observed from 2012 onwards with a peak in 2014 and affecting mainly children <1 year of age, which was the age group with the highest case fatality rates. In the two years that followed the NIP recommendation in 2014, Tdap vaccination coverage of pregnant women was low and varying from region to region.

Previous data on pertussis incidence and fatality rates in Brazil were published by Guimaraes et al.^20^ We extended this information as we included a broader time period [*i.e*. the year 2015] in our analysis of epidemiological data. Moreover, this is the first study assessing pertussis incidence observing a short period after the implementation of Tdap in the maternal immunization program in Brazil. Furthermore, the present study adds the information on hospitalizations that was lacking in the Guimaraes et al. study.^20^

Our incidence rate data are in agreement with previous studies in Brazil and other countries. In Brazil, Guimaraes et al.^20^ reported similar incidence rates for the overall population in 2012–2014. Moreover, the present study further analyzed data by age groups and found that the increase in pertussis incidence, mainly affected children aged <1 year. The Southeast region, where the largest cities are located, has the highest population density (87 people/km^2^) in the country followed by the South region (50 people/km^2^); therefore, it is reasonable to record the highest numbers of pertussis cases, hospitalizations, and deaths.^28^ Similar increase in pertussis incidence from 2007 to 2013 was reported by Torres et al.^29^ for the state of Parana, where 67.5% of pertussis cases were children <1 year of age. With respect to other countries epidemiological data, year-to-year comparisons are not feasible because of the cyclical appearance of the disease but also because of differences in the epidemiological surveillance capacity and disease confirmation criteria. Furthermore, when interpreting pertussis epidemiological data, we should take into account its evolution in cycles and the increase of the use of biological diagnosis.^30^

The epidemiological data from this study showed that nearly all deaths and hospitalizations due to pertussis in Brazil were in children <1 year of age. We found that the hospitalization rates are very high in Brazil. This might be related to disease seasonality in the country and coincides with the introduction of the DTwP-Hib-HBV to the NIP in 2012.^20^ Also, this could hinder a bias towards reporting only the more severe pertussis cases, then an underreporting of pertussis cases is also possible. For example, comparing with the US, the hospitalization rate in Brazil was approximately six or nine times higher [US respective hospitalization rates due to pertussis: 9.9% among cases reported to the national surveillance system during 2000–2016,^31^ and 6.9% in the latest provisional surveillance report^32^]. However, cross-country comparisons should be cautious, given the differences in variables affecting diseases’ epidemiology and the differences in the surveillance systems. The hospitalization rate varied by regions and exceeded 50% in children <1 year of age in all Brazilian regions, reflecting the severity of the cases. This is in line with the data of Mancaneira et al.^33^ showing that in the period 2011–2013 pertussis-related hospital admissions increased by 242% compared to 1996–2010, and most involved children <1 year of age; deaths due to pertussis had a growing temporal trend from 2011 to 2013. We found the highest mortality rate in the North and lowest in the South, in agreement with Guimaraes et al.,^20^ who recorded an overall mortality rate of 2.1%, highest in the North region (2.47) and lowest in the South (1.31). The differences in hospitalization and mortality rates between the regions may be explained by the different socioeconomic conditions, including care and access to health services and laboratory facilities for cases confirmation^34^ and sample examination,^33^^,^^35^^,^^36^^,^^37^^,^^38^^,^^39^ potentially introducing reporting bias and data incompleteness. Furthermore, the capacity of detection, investigation, and reporting of mild cases, is different between regions. The North region mainly reports severe cases while the Southeast region reports mild cases as well. This difference impacts the surveillance systems of each, affecting thus the estimating rates of lethality and hospitalization.

### Immunization in pregnancy

To the best of our knowledge, vaccination coverage of pregnant women was not previously assessed. In Brazil, the NIP has recently modified the vaccination notification protocol^40^ and this might affect the number of notifications as the health professionals have to adapt themselves to the new system; moreover different issues related to the new system’s operationalization/implementation may have emerged in the country. However, the Tdap maternal immunization coverage in the period of our study did not exceed 50% in the year after introduction and this is undoubtedly a worrying situation, considering the increased incidence of pertussis demonstrated here, affecting mainly children<1 year of age. The study of Druzian et al.,^35^ reported that 35% of pertussis cases in Mato Grosso do Sul during the decade 1999–2008, involved children not yet eligible for primary vaccination as they were <2 months of age. At that time Tdap maternal immunization was not available, which according to WHO is likely the best additional strategy for preventing pertussis in infants too young for vaccination.^10^^,^^41^ However, even with low coverage (9.2% in 2014, 40.4% in 2015) an impact may be suspected as in 2015 the study recorded a decline in all epidemiological measures assessed, in particular, the incidence rate dropped from 175.20 per 100,000 in 2014 to 64.93 in 2015.^42^ In agreement with this, a study for the state of São Paulo,^43^ reported a non-significant decline of pertussis cases in 2015, in infants <2 months of age that was attributed to the impact of maternal vaccination.

Low maternal immunization coverage has also been reported in other countries: 25.6% (95% confidence interval [CI], 24.1–26.1%) was recorded in 2015, a year after the implementation of maternal immunization in Catalonia, Spain,^44^ and 39.2% (95%CI, 33.2–45.6%) coverage in 2014 was reported in Belgium.^45^ However, in the US, coverage was gradually increasing from its introduction in 2012,^46^ to reach 53% in 2015 among the general population of pregnant women participating to the Birth Defect Study,^47^ and 53.2% in 2016 among privately insured pregnant women.^48^ In Argentina, maternal immunization coverage was 50.9%, and 67.2% in the first and second year following its introduction by the MoH in 2012, representing an increase of 16.3% (95%CI, 16.1–16.4%).^49^ Furthermore, in the United Kingdom, maternal vaccination coverage is routinely about 60%.^50^ Reasons for the low immunization rate in pregnant women include women’s lack of knowledge about pertussis disease and its possible repercussions for their children, worries over vaccine safety issues,^44^^,^^45^ insurance coverage issues, premature delivery,^51^ lack of direct recommendations from a health-care provider, and little knowledge, or lack of provision of information on immunization protocols during pregnancy to medical assistants such as midwives.^45^ Maternal immunization is a relatively new concept^52^ and pregnant women are still reluctant to take any medication during pregnancy. Also, they usually feel healthy themselves and are unaware of the immunization requirements throughout an adult’s life.^52^ Furthermore, in Brazil, pertussis is considered a child disease and its impact in newborns is underestimated. This notion has possibly contributed, with all the factors mentioned above, to the 2016 decrease (33.8%) in maternal immunization coverage. Other factors include the absence of extensive Brazilian guidelines on maternal vaccination and, possibly, the lack of related educational programs for health-care professionals and pregnant women.

### Future perspectives

Improving vaccination strategies especially for Tdap maternal immunization and vaccinations of newborns are currently among the key factors towards controlling pertussis.^53^ To improve maternal immunization coverage, vaccine promotion has been suggested, addressing safety concerns and emphasizing disease severity in infancy.^54^ Moreover, to effectively address the problem, we need to understand the main factors contributing to it; this could be answered in future studies designed to assess knowledge, attitudes, and practices related to maternal immunization among pregnant women and health-care professionals. Furthermore, we should put our efforts to improve vaccine coverage of children as well as timely administration of the vaccine, and we need to raise awareness on the severity of the disease. In case of increased incidence during the next pertussis cycle, the additional strategies of maternal immunization, cocooning, booster dose in adolescent/adults or health-care worker vaccination should be used as recommended by the WHO^41^ and by the Global Pertussis Initiative.^55^

There is a need to improve surveillance systems and laboratory capacity and access to enhance our understanding of pertussis epidemiology and provide valuable information to the research for new vaccines.^53^ Evaluating Brazil’s surveillance system, Teixeira et al.^56^ suggested improvements including integration between different health services, crisis management protocols, and increase personnel training.^56^ Lightly populated areas of the borders with limited ability to employ highly trained professionals could use high technology information surveillance networks offering real-time reporting of cases and easing communication between teams at state and national level.^56^^,^^21^ Lastly, Netto et al.^34^ suggested that Brazil’s surveillance system would benefit from a process evaluating its performance at the state level that could inform on local problems needing to be addressed.^34^ It is important to enhance the training of the health-care professionals on all aspects of their dual role as patients’ educators and as responsible for notifying pertussis cases to the surveillance systems.

### Limitations

As with any study analyzing surveillance datasets, this analysis inherits the shortcomings of the databases used. Therefore, there is a potential of bias in data derived from secondary databases due to an accurate recording while underreporting in SINAN of diseases, hospitalizations, and deaths have been raised in previous studies.^22^^,^^23^ Although Brazil has a national informatics system (DATASUS) with different databanks, it still struggles with data sub-notification and reliability on the data, there are still municipalities areas with no access to computer which could affect notifications and therefore introduce artificial differences in disease’s incidence.^43^ SIM and SINAN have shown in the past variable reporting efficiency between different regions.^27^ Studies have recorded failure in adequately reporting important disease variables in SINAN database;^27^ however, the limitations are acknowledged and since its introduction SINAN was undergone several modification to improve system’s efficacy.^57^^,^^58^ Therefore, surveillance epidemiological data require time for validation, thus 2016 epidemiological data were still under review at the time of this analysis. However, 2016 Tdap vaccination coverage data had been verified and available for analyses. Furthermore, pertussis is difficult to diagnose, especially in adults, and many cases may therefore not have been reported to authorities^30^^,^^21^^,^^59^ which means that the numbers here may well underestimate the real extent of the problem. Changes in diagnostic methods might have also affected the rate of reporting; the progressive introduction of a PCR method for diagnosis in some states as São Paulo (in 2009), Paraná and Bahia (in 2014) might have increased the number of notified cases.^20^^,^^43^^,^^60^^,^^61^ Our analysis is focused in children <4 years of age which comprised the 77% of all cases, the rest 23% contains all other ages and thus we may not draw conclusions related to other age groups of interest such as adults and young women. Furthermore, the database’s information on the vaccination status and timing were not verified with the vaccination booklet. Therefore, the children with pertussis between 1y and 4y were maybe incompletely vaccinated, or not vaccinated on time. Although this type of information is predicted to be included in the updated Brazilian vaccination notification protocol^40^ full implementation of this process will take time. At last, the surveillance system is lacking the information on the status of Tdap vaccination during pregnancy of the mothers with diseased children <1 year of age.

## Conclusions

This study’s epidemiological findings demonstrate that pertussis is still an important public health issue. A high mortality rate amongst very young children is the undesirable consequence of the increased incidence of pertussis, reflecting its seriousness. Inclusion of Tdap vaccination in the maternal immunization program in 2014 was a critical strategy, however, uptake has been slow and co-ordinated efforts and national plans are needed to increase awareness and maternal immunization coverage.

## Methods

Pertussis disease appears on the list of diseases of compulsory notification of the Health Surveillance Secretariat of the MoH. The disease is listed under the code A 37.0 as per the International Classification of Diseases, version 10 (ICD 10). In Brazil, the reporting of all suspected pertussis cases is obligatory. The health-care professionals are responsible to notify the surveillance systems through standardized electronic forms containing all relevant information, including demographics, vaccination status, hospitalizations, and death. This analysis used the “confirmed pertussis cases”^57^ (suspected cases were not available) collected between 2010 and 2015 which include laboratory, clinical or epidemiologically confirmed cases from the following nation-wide information systems of the MoH: the National Information System for Notifiable Diseases, (SINAN, *Sistema de Informação de Agravos de Notificação*),^24^ the Hospitalization Information System (SIH, *Sistema de Informações Hospitalares*),^25^ and the Mortality Information System (SIM, *Sistema de Informação sobre Mortalidade*)^26^

Furthermore, we used data collected between 2014 and 2016 from Brazil’s Unified Health System (SUS, *Sistema Único de Saúde*) on the vaccination of pregnant women from the Information system of the National Immunization Program (SI-PNI, *Sistema de Informação do Programa Nacional de Imunizações*).^62^ Because Tdap maternal immunization was introduced in late 2014 (November),^63^ we decided to analyze data from at least two years after implementation (Nov 2014–Dec 2016), although we could not include 2016 epidemiological data as these were still under review’.

### Variables and outcomes

The extracted data were a) the number of cases with pertussis infection, b) the number of hospitalizations and mortality due to pertussis, c) pertussis vaccination coverage (Tdap), d) geographical distribution of pertussis cases, and e) age at disease onset.

Outcomes included a) epidemiological data by geographical region, year, and age group, and b) Tdap coverage in pregnant women.

### Statistical analysis

The programs *Excel, Medcalc*, and SPSS version 20.0 were used. The chi-square test was used to compare proportions, and the Student’s t-test for independent samples to compare means. In all analyses, a level of significance of *p* < .05 was considered statistically significant.

## Figures and Tables

**Table:** 

CI	confidence interval
DTP	diphtheria tetanus pertussis vaccine
DTwP-Hib-HBV	diphtheria tetanus whole pertussis - H. influenza type B - hepatitis B vaccine
MoH	Ministry of Health
NIP	National Immunization Program
SIH	Sistema de informações Hospitalares
SIM	Sistema de Informação sobre Mortalidade
SUS	Sistema Único de Saúde
SINAN	Sistema de Informação de Agravos de Notificação
SI-PNI	Sistema de Informação do Programa Nacional de Imunizações
Tdap	diphtheria tetanus acellular pertussis vaccine
US	United States

## Highlights

- Our recent data showed an increase of pertussis incidence between 2010 and 2014 but a decrease in 2015, confirming a pertussis cycle as in other countries
- Most cases and deaths occurred in children <1 year of age
- Maternal vaccination coverage was around 40% in 2015 but decreased in 2016, correlating with the decrease of the pertussis cycle and probably with a decrease of information concerning the severity of the disease

## Data statement

This study focused on gathering evidences related to pertussis burden of disease before and after Tdap vaccine introduction in the National Immunization Program. The epidemiological data analysis is focused to complement the literature data, using the Ministry of Health public databases (SINAN, Sistema de Informação de Agravos de Notificação; SIH, Sistema de Informações Hospitalares; and SIM, Sistema de Informação sobre Mortalidade), demonstrating the importance of new preventive strategies against pertussis in vulnerable populations (in special, children <1 year of age).
